# Development of a prediction model of severe reaction in oral food challenge

**DOI:** 10.1186/2045-7022-5-S3-O21

**Published:** 2015-03-30

**Authors:** Sugiura Shiro, Matsui Teruaki, Sasaki Kemaru, Nakagawa Tomoko, Nakata Joon, Kando Naoyuki, Ito Komei

**Affiliations:** 1 Department of Allergy, Aichi Children’s Health and Medical Center, Obu, Japan

## 

Recently, oral food challenge testing (OFC) is performed not only to confirm a diagnosis of food allergy but also to evaluate the threshold dose and severity of anaphylactic reaction. We have proposed a new scoring system (Anaphylaxis Scoring Aichi: ASCA) for a quantitative evaluation of anaphylactic reaction observed in an OFC, and TS/Pro (Total Score of ASCA / cumulative protein dose) to represent the overall severity of reaction (Ito K; EAACI 2013, Milan).

The purpose of this study was to identify the clinical factors contributing to high TS/Pro, and to develop a prediction model for severe reaction.

*Development Phase:* Patients resulted in positive OFC to egg (n=198), milk (n=106) and wheat (n=105) performed during April 2012 to May 2013(egg, milk), to November (wheat). Validation phase: All patients who performed an OFC, irrespective of the result, during June 2013 to October 2013(egg), June 2013 to March 2014(milk), April 2011 to March 2012(wheat). Patients lacking laboratory data within 6 month before OFC were excluded.

“Severe reaction” was defined as the TS/Pro higher than the median score of each allergen (egg: 30, milk: 75, wheat: 50). Multivariate logistic regression analysis was done to predict the severe reaction (criterion variable), using allergen-specific IgE titer (ImmunoCAP, sIgE class) and some clinical factors as explanatory valuables.

sIgE class and some clinical factors (total IgE, age, history of anaphylaxis, complete avoidance of the allergen) were independently associated with severe reaction, with some variation between allergens. Based on the sIgE class and the proportion of each factor, we made a simple prediction model. In the development phase, the prediction model for egg, milk and wheat showed good predictive value by ROC analysis; AUC 0.83, 0.84 and 0.90, respectively, which were better than simply applying the sIgE class of each allergen. Good prediction was reproduced in the validation phase; AUC 0.84, 0.80 and 0.87, respectively.

The prediction model was useful to decide the indication of OFC and to make a safer OFC protocol.

